# Every Beat You Take—The Wilms′ Tumor Suppressor WT1 and the Heart

**DOI:** 10.3390/ijms22147675

**Published:** 2021-07-18

**Authors:** Nicole Wagner, Kay-Dietrich Wagner

**Affiliations:** CNRS, INSERM, iBV, Université Côte d’Azur, 06107 Nice, France

**Keywords:** Wilms’ tumor suppressor 1 (Wt1), heart, cardiac development, coronary vessel formation, transcriptional regulation, cardiac malformation, epicardium, epicardial derived cells (EPDCs), epithelial mesenchymal transition (EMT), cardiac cell fate, regeneration

## Abstract

Nearly three decades ago, the Wilms’ tumor suppressor Wt1 was identified as a crucial regulator of heart development. Wt1 is a zinc finger transcription factor with multiple biological functions, implicated in the development of several organ systems, among them cardiovascular structures. This review summarizes the results from many research groups which allowed to establish a relevant function for Wt1 in cardiac development and disease. During development, Wt1 is involved in fundamental processes as the formation of the epicardium, epicardial epithelial-mesenchymal transition, coronary vessel development, valve formation, organization of the cardiac autonomous nervous system, and formation of the cardiac ventricles. Wt1 is further implicated in cardiac disease and repair in adult life. We summarize here the current knowledge about expression and function of Wt1 in heart development and disease and point out controversies to further stimulate additional research in the areas of cardiac development and pathophysiology. As re-activation of developmental programs is considered as paradigm for regeneration in response to injury, understanding of these processes and the molecules involved therein is essential for the development of therapeutic strategies, which we discuss on the example of WT1.

## 1. Introduction

The Wilms’ tumor 1 (WT1) gene was originally identified based on its mutational inactivation in Wilms’ tumor (nephroblastoma) [1,2,3]. This first discovery of WT1 as the responsible gene in an autosomal-recessive condition classified it as a tumor-suppressor gene. Mutations of WT1 were associated with the development of kidney tumors and urogenital defects. However, later it became clear that mutations of WT1 only occur in a low frequency in nephroblastoma [4] and that most nephroblastomas [5] express high levels of WT1. Based on the overexpression of WT1 in leukemia and most solid cancers (reviewed in [6,7] and its cancer-promoting functions in the tumor stroma [8], WT1 is nowadays considered as an oncogene and attractive candidate for cancer therapy.

WT1 encodes a zinc finger transcription factor and RNA-binding protein [9,10,11,12]. As a transcriptional regulator, it can either activate or repress various target genes. Thus, WT1 influences cellular differentiation, growth, apoptosis, and metabolism. WT1 exists in multiple isoforms. Alternative splicing of exon 5 and exon 9 gives rise to major isoforms. Splicing of exon 9 generates the KTS isoforms, which either include or exclude three amino acids lysin, threonine, and serin (KTS) between zinc fingers 3 and 4 of the protein [13]. Although the majority of WT1 proteins are in the nucleus, some are present in the cytoplasm, located on actively translating polysomes. WT1 isoforms shuttle between the nucleus and cytoplasm [14]. The complexity of WT1 is further enhanced by post-translational modifications and a plethora of binding partners. WT1 directs the development of several organs and tissues, among them the heart.

The heart develops mostly from embryonic mesodermal germ layer cells and to some extent from ectoderm-derived cardiac neuronal crest (cushions of the outflow tract). The cardiogenic mesoderm differentiates into proepicardial, endocardial, and myocardial cells. The epicardium is formed from a subset of the proepicardial cells. Proepicardial cells also contribute subepicardial cells, interstitial fibroblast, pericytes, and a subset of the endothelial cells of the coronary vessels. The inner lining of the heart tube is formed by endocardial cells. The vertebrate heart forms as two concentric epithelial cylinders of myocardium and endocardium separated by an extended basement membrane matrix commonly referred to as cardiac jelly. The primitive heart tube is formed at embryonic day 8.5 (E8.5) in the mouse [15]. The primitive tube elongates and undergoes rightward looping. Further remodeling of the heart involves formation and expansion of the chambers, and formation of valves and septa, resulting in a heart with two atria and two ventricles [16]. The heart is the first organ to develop and is already functional at an early stage of fetal development, in line with its essential role for the distribution of oxygen and nutrients and removal of waste products and carbon dioxide. Several excellent reviews have already described cardiac development in detail [17,18,19,20,21,22]. Thus, we focus here only on the role of Wt1. Wt1 expression was first observed in a transitory cluster of cells—the proepicardium and the coelomic epithelium at E9.5. Wt1-expressing proepicardial cells contact the dorsal wall of the heart from which the proepicardial cell migrate over the myocardium of the heart tube to form the epicardial layer by E12.5 [23,24]. This view has been challenged recently by the detection of a common progenitor cell population of epicardium and myocardium using single-cell RNA sequencing [25]. How these common progenitors might migrate during cardiac development is currently an open question.

A proportion of epicardial cells undergoes epithelial-to-mesenchymal transition (EMT), which induces the formation of epicardial-derived cells (EPDCs), a population of multipotent mesenchymal cardiac progenitor cells, which might differentiate into cardiomyocytes, fibroblasts, smooth muscle, and endothelial cells [26,27,28], which is discussed in detail later. First indications for the indispensable role of Wt1 in heart homeostasis came from the observations made in Wt1 knockout embryonic mice which died at mid-gestation due to cardiac malformations [29].

Here, we review the history of investigations characterizing the role of WT1 (i) in cardiac development, (ii) in cardiac disease and regeneration, and (iii) in different cardiac cell types and transcriptional regulatory mechanisms. We indicate emerging notions and point out problems and promises in the field of development of therapeutic strategies for cardiac repair.

## 2. WT1 in Heart Development

Nearly thirty years ago, Armstrong and colleagues, using in situ mRNA hybridization, observed Wt1 expression in the differentiating heart mesothelium of the mouse embryo at embryonic day 9 [23]. In the same year, the group of Jaenisch introduced a mutation into the murine *Wt1* gene by gene targeting in embryonic stem cells. The embryos homozygous for this mutation died between days 13 and 15 of gestation. Besides the lack of kidney and gonad formation in *Wt1* mutant mice, the authors observed a severe heart hypoplasia with thinned right ventricular walls, a rounded apex, and a reduction of size of the left ventricles, signs of congestive heart failure, suggesting that cardiac malfunction was the cause of early embryonic death [29]. As Wt1 has been described before only to be expressed in the epicardium, but has not yet been observed in the myocardium, it remained unclear whether these features of cardiac malformation were due to primary defects in the myocardial tissue or a consequence of disturbed development in other tissues. A more detailed view on Wt1 expression during murine heart development was achieved using a lacZ reporter gene inserted into a YAC (yeast artificial chromosome) construct which demonstrated Wt1 expression in the early proepicardium, the epicardium, and subepicardial mesenchymal cells (SEMCs) throughout development. In Wt1-deficient animals, the epicardium did not form correctly, which results in disruption in the formation of the coronary vasculature, leading to pericardial bleeding and midgestational death of the embryo. Complementation of *Wt1* null embryos with a human *WT1* transgene rescued both embryonic heart defects and midgestational death, confirming that indeed heart failure causes the death of Wt1-deficient embryos [24].

Wt1-expressing cell types during heart development in different species are summarized in Table 1 and further described below. Of note, expression of Wt1 is limited to a subset of the identified cells. Functional differences between Wt1-expressing cells and the Wt1-negative counterparts remain mostly unknown at present.

Studies in birds confirmed the expression of Wt1 in epicardium- and epicardial-derived cells (EPDCs) during embryonic development [31]. Using normal avian and quail-to-chick chimeric embryos, the origin and fate of Wt1-expressing EPDCs were later described and the effects of epicardial ablation on cardiac development investigated [28]. Wt1-expressing EPDCs were found to populate the subepicardial space and to invade the ventricular myocardium. Upon differentiation in smooth muscle and endothelial cells, Wt1 expression decreased in EPDCs. Undifferentiated EPDCs continued to express Wt1 and invaded the ventricular myocardium and the atrio-ventricular (AV) valves. Disruption of normal epicardial development either by proepicardial ablation or block reduced the number of invasive Wt1-positive EPDCs, and provoked anomalies in the coronary vessels, the ventricular myocardium, and the AV cushions. In addition to Wt1, EPDCs express retinaldehyde-dehydrogenase (Raldh) 2 [38,39]. It had been demonstrated that in humans WT1 transcriptionally regulates the retinoic acid receptor alpha (*RAR-α*) gene [40]. Transcriptional target genes of WT1 with relevance in the heart are summarized in Table 2 and discussed below.

The phenotype of the WT1-deficient mice further resembled that of retinoic acid (RA)-depleted mice. Depletion of RA from the diet is known to severely disturb heart development, causing hypoplasia of the ventricles [41]. The authors suggested therefore that Wt1 maintains the EPDCs in an undifferentiated, RA-producing state to contribute to ventricular myocardium compaction in the development of the myocardial wall [28]. Availability of retinoic acid during cardiac development is mediated by Raldh2. It has been shown that Wt1 transcriptionally activates Raldh2 [42]. Pericardium and sinus horn formation are coupled and are based on the expansion and exact temporal release of pleuropericardial membranes (PPM) from the underlying subcoelomic mesenchyme. Wt1-deficient mouse embryos displayed a failure to form myocardialized sinus horns and a loss of Raldh2 expression in the subcoelomic mesenchyme, pointing to a crucial role of Wt1 and downstream Raldh2/RA signaling in sinus horn development [43]. Furthermore, Wt1-mutant mice were shown to display unilateral partial PPM absence in the dorsomedial region. Failure of PPM release affects the closure of the remaining communication area between pericardial and pleural cavities, the bilateral pericardioperitoneal canals (PPCs), which is disturbed in Wt1-deficient embryos, leading to pleuropericardial communication and lateralization of the cardinal veins [44]. The group of Muñoz -Chapuli suggests that the proepicardium is an evolutionary derivative of the primordium of an ancient external pronephric glomerulus, initially based on the epicardial development in lampreys (Petromyzon), the most primitive living lineage of vertebrates [45]. Employing chick proepicardium, they propound that Wt1 could repress the nephrogenic potential of the proepicardium, while at the same time promote nephrogenesis in the intermediate mesoderm. This paradoxical function could be explained by the dual role of Wt1, which promotes mesenchymal to epithelial transition (MET) in the kidney and EMT in the epicardium [46], through a mechanism known as chromatin flip–flop [47]. Promotion of EMT in the developing epicardium and MET in the growing kidney is not only reflected by morphological cellular changes, but also differential expression of podocyte markers. In their study, the authors focused on podocalyxin, known to be transcriptionally regulated by Wt1, and to be activated by Wt1 in kidney podocytes [48], which they found in contrast to be upregulated in Wt1-deficient epicardium [46]. To further strengthen this theory it appears interesting to investigate the expression of other Wt1 transcriptional targets in kidney MET and epicardial EMT, such as nephrin [49], nestin [50], and podocin [51].

In addition, the relation between Wt1-expressing epicardial derivatives and the development of compact ventricular myocardium has been investigated. The differences in myocardial architecture specifically between the right ventricle (RV) and the left ventricle (LV) in association to epicardial formation and distribution of Wt1-expressing cells were studied. The authors demonstrated that the RV is less densely and later covered by the epicardium than the LV. They also observed that compact myocardial layer formation occurred in parallel with the presence of Wt1-expressing cells and was more pronounced in the LV than in the RV, and within the RV more accentuated in the postero-lateral wall than in the anterior wall, which might explain the lateralized differences in ventricular morphology of the heart [52]. The same group was able to identify a function of the epicardium in cardiac autonomic nervous system modulation, essential for proper cardiac activity by altering heart rate, conduction velocity, and force of contraction. They revealed expression of neuronal markers in the epicardium during early cardiac development, notably of tubulin beta-3 chain (Tubb3), which was colocalized with Wt1 in epicardium and the nervous system, neural cell adhesion molecule (Ncam), and the β2 adrenergic receptor (β2AR). Adrenaline (epinephrine), a catecholamine, is known to modulate heart rate, velocity of conduction, and contraction strength in the heart through its binding to β2AR. Inhibition of the outgrowth of the epicardium abolished the response to adrenaline administration, indicating that the epicardium is necessary for a normal response of the heart to adrenaline during early cardiac development [53]. This report further confirmed a role of Wt1 in neural function, as suggested by several studies [23,54,55,56,57,58,59].

In zebrafish, two orthologues of *wt1* have been described: *wt1a* [60,61] and *wt1b* [62]. Both of them were found to be expressed in adult zebrafish hearts, but exhibited a differential expression level in other organs, as well as a differing temporal patterning during development, suggesting distinctive functions during zebrafish development [62]. During zebrafish cardiac development, Wt1 is required for the proper development of the proepicardial organ and epicardial lineage [30]. A later study proposed that Wt1-interacting protein (Wtip), a protein identified as a Wt1-interacting partner by a yeast two-hybrid screen [63], signals in conjunction with WT1 for proepicardial organ specification and cardiac left/right asymmetry in the zebrafish heart [64]. Two main cardiac cell types were suggested to be involved in zebrafish heart regeneration using ex vivo cultures: epicardial cells, displaying a larger, prismatic morphology and Wt1/Gata4 (Gata-binding protein 4) expression, and endocardial small, rounded cells, positive for Nfat2 (nuclear factor of activated T-cells 2) and Gata4 [65].

**Table 2:** WT1-target genes related to cardiac development and disease.

Gene	Reference
Insulin like growth factor 1 receptor (IGF-1-R)	[66]
Epidermal growth factor receptor (EGFR)	[67]
Retinoic acid receptor alpha (RAR- α)	[40]
Retinaldehyde-dehydrogenase (Raldh) 2	[42]
Insulin receptor (IR)	[68]
Paired box gene 2 (Pax2)	[69]
Platelet-derived growth factor A (PDGFA)	[70,71]
Early growth response protein 1 (EGR-1)	[72]
Insulin like growth factor 2 (IGF-2)	[73]
Transforming growth factor beta (TGF-β)	[74]
Colony-stimulating factor-1 (CSF-1)	[75]
Syndecan 1	[76]
Midkine	[77]
Vitamin D receptor (Vdr)	[78,79]
Podocalyxin	[48]
Nephrin (Nphs1)	[49]
Podocin (Nphs2)	[51]
Tyrosinkinase receptor (Trk)B	[32]
Nestin	[50]
Erythropoietin (EPO)	[80]
α4 Integrin	[81]
Vascular endothelial growth factor (VEGF)	[82,83]
Vascular endothelial growth factor receptor (Vegfr) 2	[84,85]
ETS proto-oncogene (ETS)-1	[84]
Snail (Snai1)	[86]
Slug (Snai2)	[87]
E-Cadherin	[86,88]
VE-Cadherin	[89]
Coronin1B	[90]
Cxcl10 (C-X-C Motif Chemokine Ligand 10)	[91]
Ccl5 (C-C Motif Chemokine Ligand 5)	[91]
Interferon regulatory factor (Irf)7	[91]
c-Kit (tyrosine-protein kinase KIT)	[8]
Pecam-1 (platelet and endothelial cell adhesion molecule 1)	[8]
Telomere repeat binding factor (Trf) 2	[92]
Bone morphogenetic protein (Bmp) 4	[93]

## 3. WT1 in the Adult Heart and Cardiac Pathologies

Already in 1994, Wt1 transcripts were detected by Northern blot in adult rat heart tissues [94]. Whether modifications in Wt1 expression occur under pathophysiological conditions and which cell types express the protein remained open questions. Our group was the first to demonstrate that Wt1 is a useful early marker of myocardial infarction [95], a finding later confirmed by others [96,97,98]. We focused on the de novo Wt1 expression in the coronary vasculature of the ischemic myocardium. As Wt1 is essential for normal growth of the heart during development, we originally reasoned that it might also play a role in adult cardiac hypertrophy. To test this hypothesis, we analyzed the expression of Wt1 in normal hearts and in the hypertrophied left ventricles of spontaneously hypertensive rats (SHRs), with activation of the renin–angiotensin system by transgenic (over) expression of human renin and angiotensinogen genes, and with postinfarct remodeling of the heart after ligation of the left coronary artery (LAD). Interestingly, we detected an over two-fold increase of cardiac Wt1 mRNA expression after LAD ligation, but no differences for the two hypertrophy models compared to controls. Further experiments using LAD ligation demonstrated a rapid increase of cardiac Wt1 levels already 24 h after LAD ligation, which remained elevated for nine weeks following the ischemic injury. Strikingly, in addition to its expression in the epicardium, we observed Wt1 localized to the coronary vessels in proximity to the infarcted tissue. Coronary vessels of non-infarcted animals did not express Wt1. Wt1 was expressed in endothelial as well as in vascular smooth muscle cells in the border zone of infarcted tissues. We confirmed this finding also in human cardiac ischemic tissues (unpublished results). Interestingly, WT1 expression could also be detected in healthy adult human myocardium by others [99]. Colocalization of Wt1 with proliferating cell nuclear antigen (PCNA) and vascular endothelial growth factor (VEGF) suggests a role of Wt1 in the proliferative response of the coronary vasculature to cardiac hypoxia [95]. In a following study, we were the first to demonstrate that Wt1 expression is indeed triggered by hypoxia, which involves transcriptional activation of the Wt1 promoter by the hypoxia inducible factor 1 (HIF-1) [100]. Later studies confirmed our finding that Wt1 is a hypoxia-regulated gene [83,101]. Interestingly, it had been demonstrated that ischemia in vivo (through myocardial infarction in mice) or in vitro (hypoxia exposition of epicardial human explants) induced an embryonic reprogramming of the epicardial compartment, involving migration of epicardial-derived stem cell marker c-Kit expressing Wt1-positive cells which contributed to re-vascularization and cardiac remodeling [102]. As we identified c-Kit as a transcriptional target of Wt1 in the context of vascular formation [8], it seems conceivable that mobilization of c-Kit precursor cells represents one mechanism of Wt1-mediated cardiac neovascularization after ischemia. We further identified the telomere repeat-binding factor (Trf) 2 to be regulated by Wt1 [92]. Down-regulation of Trf2 has been demonstrated to provoke cardiomyocyte telomere erosion and apoptosis, linking telomere dysfunction to heart failure [103].

Thymosin β4 (Tβ4), a 43-amino-acid G-actin-sequestering peptide which is expressed in the embryonic heart and implicated in coronary vessel development in mice [104], has been shown to activate cardiac regeneration through stimulation of the expression of embryonic developmental genes in the adult epicardium, leading to de novo coronary vessel formation after myocardial infarction. However, a significant increase could only be reported for Vegf, Vegfr2, and TGFβ levels, whereas Wt1 levels were not significantly altered 24 h after MI compared to vehicle-treated animals [105]. A later study additionally revealed that adult Wt1^+^ GFP^+^ EPDCs cells obtained through Tβ4 priming and myocardial infarction are a heterogeneous population expressing cardiac progenitor and mesenchymal stem markers that can restore an embryonic gene program, but do not revert entirely to adopt an embryonic phenotype [106].

First suspicions for a role of Wt1 in human cardiac pathologies originated in 2004, with a case report from an adult XY karyotype patient with a N-terminal *WT1* missense mutation presenting a very unusual phenotype: ambiguous genitalia, but normal testosterone levels, absence of kidney disease, and an associated congenital heart defect [107]. Later, a role for WT1 in some cases of congenital diaphragmatic hernia associated with the Meacham syndrome phenotype had been suggested [108]. Meacham syndrome is a rare sporadically occurring multiple malformation syndrome characterized by male pseudo-hermaphroditism with abnormal internal female genitalia, complex congenital heart defects, including hypoplastic left hearts, and diaphragmatic abnormalities [109]. In a number of Meacham syndrome patients, heterozygous missense mutations in the C–terminal zinc finger domains of WT1 could be identified, suggesting that at least some cases displaying phenotypes of Meacham syndrome are caused by mutations at the *WT1* locus [108]. We reported the case of a 4-month-old girl, who presented with end-stage renal disease, nephroblastomatosis, thrombopenia, anemia, pericarditis, and cardiac hypertrophy accompanied by severe hypertension. Sequence analysis identified a heterozygous nonsense mutation in exon 9 of *WT1*, which leads to a truncation of the WT1 protein at the beginning of zinc finger 3 [110]. WT1 is a transcriptional regulator of erythropoietin, which might explain the persistent anemia in this patient [80]. Evolution over time showed severe and resistant high blood pressure, despite multi-drug therapy and bilateral nephrectomy, which did not result in the normalization of the blood pressure values. Acute episodes of high blood pressure were associated with cardiogenic shock and anemia. The little patient showed a severe concentric myocardial hypertrophy, with moderate signs of heart failure and intermittent pericarditis [110]. Still awaiting kidney transplantation, the child died due to myocardial infarction at the age of five years. Later, another case of cardiac pathology in a patient with a *WT1* mutation was reported: A 46, XY phenotypic male patient with isolated nephrotic syndrome, end-stage renal disease, and hypertension, presented at the age of 6.3 years. A mutation in exon 8 of the *WT1* gene was identified. After starting hemodialysis, manifestations of hypertension and renal failure improved, but he died at 6.8 years of age as a result of heart and respiratory failure [111]. Monozygotic twins with congenital nephrotic syndrome caused by a *WT1* mutation have been reported to have died due to sepsis and extensive thrombosis of central venous system and sepsis and sudden heart failure at ages 23 weeks/13.5 months, respectively [112]. WT1 misexpression has been reported in autopsy findings from two human fetuses, displaying congenital pulmonary airway malformation, bilateral renal agenesis, and congenital heart defects [113]. Shortly after, re-evaluation of autopsy data from fourteen additional fetuses with combined renal agenesis and cardiac anomalies revealed abnormalities of Wt1 expression, mostly in liver mesenchymal cells. As WT1 is widely expressed in mesothelium, it had been suggested that the defects could be caused by abnormal function of mesenchyme derived from mesothelial cells [114]. WT1 is further expressed in cardiac angiosarcomas, which is the most common malignant neoplasm of the heart in adults. As other primary cardiac malignancies such as synovial sarcoma, leiomyosarcoma, and unclassified sarcomas are frequently negative for WT1, this finding might be helpful for differential diagnosis. It further confirms the implication of WT1 in vascular formation [115].

Interestingly, it has been shown recently using patient biopsies that the thickening of the epicardium and migration of Wt1-positive EPDCs contributes to atrial fibro-fatty infiltration, a source of atrial fibrillation. Employing Wt1 genetic lineage mouse lines, the authors showed that adult EPDCs maintain an adipogenic potential in the epicardial layer and can shift to a fibrotic phenotype in response to distinct stimuli, identifying the epicardium as a central regulator of the balance between fat and fibrosis accumulation [116]. Additionally, the expression of TGFβ1 and FGFs (fibroblast growth factors) by EPDCs has been suggested to contribute to the pathogenesis of myocardial fibrosis, apoptosis, arrhythmias, and cardiac dysfunction in a mouse model of arrhythmogenic cardiomyopathy (ACM) [117].

## 4. WT1 in the Heart–Focus on Different Cell Types and Regulatory Mechanisms

Table 3 summarizes WT1-expressing cell types in the adult heart. Reported functions and regulatory mechanisms are discussed below.

Although a relationship between Wt1 and myocardial blood vessel development had already been suggested [24,28], it remained unclear whether Wt1 is indeed necessary for normal vascularization of the heart. Coronary vessel formation is organized through a series of tightly regulated events. The epicardial cells undergo an epithelial-to-mesenchymal transition [122,123,124] to become subepicardial mesenchymal cells. The subepicardial mesenchymal cells then migrate into the myocardium, where they differentiate into endothelial cells, smooth muscle cells, and perivascular fibroblasts of the coronary vessels [124,125]. Further steps in coronary vessel formation include stabilization of the newly formed vessels and remodeling to connect the vessels to the main coronary arteries, originating from the aorta (reviewed in [126]). In contrast to this classical view, mostly lineage tracing experiments suggested an important contribution of sinus venosus-derived endothelial cells [127,128] or the endocardium [129] for cardiac vessel endothelial cells, which has been questioned later [130] (reviewed in [131,132]).

In addition to the epicardium, we clearly observed nuclear Wt1 protein expression in the coronary vessels of mouse embryos at E12.5, E15.5, and E18.5. Notably, we detected endogenous Wt1 protein but not reporter gene activity. Wt1-deficient embryos (E12.5) failed to form subepicardial coronary vessels. To identify candidate target genes of Wt1 in the process of coronary vessel formation, we performed a transcriptome analysis of differentially expressed genes from hearts of wild-type and *Wt1*-deficient mice. One of the genes found to be differentially expressed was *Ntrk2*, the gene encoding for the tyrosine kinase type B receptor (TrkB) [32]. TrkB is a tyrosine kinase receptor with high affinity for brain-derived neurotrophic factor (BDNF) and neurotrophin 4/5 (NT4/5) (reviewed in [133]). A role for BDNF signaling in coronary blood vessel formation had emerged based on the observation that BDNF-deficient mice displayed abnormal myocardial vessel formation due to endothelial cell apoptosis [134]. TrkB and Wt1 co-localized in the epicardium and the coronary vessels of mouse embryonic hearts at E12.5. TrkB expression was absent from Wt1-deficient embryonic hearts. TrkB-deficient mouse embryos revealed a reduction of coronary vessel formation along with enhanced apoptosis. In addition to these lines of evidence which suggested that *Ntrk2*, the gene encoding the TrkB neurotrophin receptor, represents a target of Wt1 in the process of myocardial vascularization, molecular approaches employing transient transfections, Dnase1 footprint analyses, and electrophoretic mobility shift assays helped to identify a binding site for Wt1 in the *Ntrk2* promoter. This binding site was necessary for transgenic expression of a lacZ reporter in the developing myocardial vasculature and other known sites of Wt1 expression as the gonads and the coelomic epithelium. Activation of TrkB expression by Wt1 appears therefore to be a critical step for the proper development of the coronary vessels [32]. Another protein that is expressed by newly forming vessels is the intermediate filament protein nestin [135]. We could demonstrate the regulation of nestin by Wt1; nestin further colocalized with Wt1 in the epicardium and the forming coronary vessels, and was undetectable in Wt1 knockout hearts [50]. Nestin has been found to be highly expressed in proangiogenic capillaries after myocardial infarction and has been proposed to play a role in remodeling the cytoskeleton of cells in the human postinfarcted myocardium [136]. Moreover, the transmembrane cell adhesion molecule α4 integrin has been identified to be a transcriptional target of Wt1 in cardiac development [81]. α4-integrin-deficient mouse embryos display epicardial and coronary vessel formation defects, similar to those observed in Wt1 knockout embryos [137]. The transcriptional activation of the *α4 integrin* gene by Wt1 could therefore be an additional regulatory mechanism for the formation of the epicardium. We further identified the major podocyte protein nephrin as a transcriptional target of Wt1 [49]. Nephrin is not only required for kidney podocyte function [138], but also is crucial for cardiac vessel formation during development. We found nephrin to be expressed during human and mouse cardiac development. Nephrin-deficient mice displayed epicardial defects, a disturbed coronary vessel formation, and an increased apoptosis predominantly in the developing epicardium. Direct interaction of nephrin with the low affinity neurotrophin receptor p75NTR and subsidiary upregulation of p75NTR are critically involved in the cardiac phenotype of nephrin-deficient embryos [139]. Cardiac abnormalities have been reported in FinMajor nephrin mutation patients, which presented mainly with mild cardiac hypertrophy [140,141,142]. Another protein critically involved in cardiac vessel formation, which is transcriptionally regulated by Wt1, is the transcription factor Ets-1 [84]. Like Wt1, Ets-1 deficiency results in a failure of epicardial differentiation, a disturbed coronary vessel formation, and myocardial defects [143]. Recently, extracardiac septum transversum/proepicardium (ST/PE)-derived endothelial cells have been shown to be required for proper coronary vascular morphogenesis. Conditional deletion of Wt1 from both, the ST/PE and the endothelium disrupted embryonic coronary transmural patterning, leading to embryonic death. The ST/PE contributes a significant fraction of cells (≈20%) to the coronary endothelium during embryogenesis required for proper coronary vascular development [144].

Using Wt1^GFPCre^ and inducible Wt1^CreERT2^ mouse lines, the group of William Pu suggested in 2008 that Wt1-expressing epicardial cells contribute to the cardiomyocyte lineage during normal heart development. Wt1 epicardial cells located on the heart at E10.5–E11.5 differentiated in vivo into fully functional cardiomyocytes, as evidenced ex vivo by spontaneous contractility and calcium oscillations with kinetics, amplitude, and frequency characteristic of cardiomyocytes [26]. Moreover, in 2008, Cai and colleagues reported the identification of a cardiac myocyte lineage that derives from the proepicardial organ. These T-box transcription factor (Tbx) 18-expressing progenitor cells migrated onto the outer cardiac surface to form the epicardium, and then contributed to myocytes in the ventricular septum and the atrial and ventricular walls [145]. Shortly after, Christoffels et al. showed that Tbx18 is expressed in left ventricular and interventricular septum cardiomyocytes independent of a epicardial contribution [146]. Both groups used independent Tbx18 Cre knock-in lines, which differed considerably in sensitivity and specificity. The results of the Tbx18 reporter system used by Christoffels et al. correspond to endogenous Tbx18 expression data reported earlier [147]. Studying in detail the migration and differentiation of epicardium-derived cells, the group of Poelman already observed in 1998 that EPDCs migrated to the subendocardium, myocardium, and atrioventricular cushions. The functional role of these novel EPDCs remained however unclear [148]. Employing chick proepicardial explant cultures, it had further been demonstrated that proepicardial cells were able to differentiate into cardiac muscle cells in vitro, reflecting the pluripotency of the pericardial mesoderm [149]. In 2011, a reactivation of *Wt1* expression after myocardial infarction resulting in cardiomyocyte restitution has been proposed. Using Wt1^CreEGFP^ and inducible Wt1^CreERT2^ lines, the authors observed only a mild increase in Wt1 expression upon cardiac injury and no initiation of cardiac vessel formation, which is in contrast to our previous findings [95]. Only by using thymosin β4 (Tβ4) priming which had before been reported to initiate expression of embryonic developmental genes in the epicardium [105], a significant reactivation of Wt1 expression after myocardial infarction was achieved resulting in the appearance of some Wt1-positive cardiomyocytes in the border zone of the infarcted area. These findings suggest a contribution of Wt1-positive EPDCs to the myocardium after myocardial infarction in the artificial setting of thymosin β4 priming before infarction [119]. However, co-authors from this study showed one year later, using the epicardium genetic lineage tracing line Wt1^CreERT2/+^ and double reporter line Rosa26^mTmG/+^, that epicardial cells do not differentiate into cardiomyocytes following myocardial infarction and Tβ4 treatment. Their study clearly raises cautions regarding a potential clinical use of Tβ4 with the goal to increase cardiac repair [150]. An additional Wt1 Cre using a BAC clone has been described, which again gave different results. Without using Tβ4, the authors observed a significant increase of Wt1 expression and proliferation in the epicardium shortly after myocardial infarction, leading to the formation of a Wt1-lineage-positive subepicardial mesenchyme until two weeks post-infarction. These mesenchymal cells were shown to contribute to the fibroblast population, myofibroblasts, and coronary endothelium in the infarct zone, a few of them later also differentiated into cardiomyocytes [118]. C. Rudat and A. Kispert undertook a substantial effort to identify Wt1-expressing cardiac cell types and to clarify the contribution of Wt1-expressing progenitor cells to differentiated cardiac cells. Using in situ hybridization and immunofluorescence, they revealed expression of Wt1 mRNA and protein not before E9.5 in the (pro)epicardium and endothelial cells throughout development. They further determined that neither Wt1^CreEGFP^ nor Wt1^CreERT2^** lineage tracing systems are reliable epicardial fate-defining approaches due to ectopic recombination and poor recombination efficiency. Endogenous expression of Wt1 in the endothelium and eventually the myocardium in the developing heart eliminates Wt1-based Cre lines to trace the epicardial contribution to myocardial, and endothelial cells in the murine heart. The proposed epicardial origin of myocardial and endothelial cells in the heart using Wt1-based Cre/loxP lines appears therefore not justified [35]. Accordingly, Wt1 had already been excluded from epicardial cell fate mapping approaches in zebrafish due to its non-epicardial expression in the fish hearts [151]. A population of adult cardiac resident colony forming unit fibroblasts (cCFUs), most likely originating from the proepicardium/epicardium has been identified, giving rise mainly to cellular components of the coronary vasculature. A differentiation into cardiomyocytes in vivo could, however, not be supported [152]. Our group found Wt1 to be expressed in cardiomyocytes during development (first time point studied E10.5) and throughout lifespan. The number of Wt1-positive cardiomyocytes as well as the individual cellular intranuclear Wt1 expression decreased during development and was very low in adulthood, but we propose that low levels of Wt1 expression are sufficient to maintain a cardiac progenitor subset from terminal differentiation. Myocardial infarction strongly up-regulated Wt1-expressing cardiomyocytes, as well as individual nuclear Wt1 expression in cardiomyocytes. Interestingly, in contrast to the expression pattern in epicardial cells, Wt1 was expressed in a speckled manner in cardiomyocytes [37], suggesting the presence of the Wt1 + KTS variant [153]. We detected Wt1+ cardiomyocytes already 48 h after myocardial infarction. Given the distance to the epicardium and the differing expression pattern of Wt1, speckled in the nucleus in cardiomyocytes versus diffuse nuclear expression in epicardial cells, it is unlikely that these cardiomyocytes are epicardium-derived [37]. It has been shown that de novo cardiomyocytes arise in adjacent areas of a myocardial infarction [154,155]. This could support cardiac tissue regeneration by Wt1 reactivation in response to stimuli as hypoxia/inflammation, inducing progenitor cell proliferation and cell survival. We observed that upon cardiac differentiation of mouse embryonic stem cells (mESCs), Wt1 expression increased. Overexpression of Wt1 in mESCs reduced phenotypic cardiomyocyte differentiation in vitro keeping the cells in a more progenitor-like stage [37]. Recently, a common progenitor pool of the epicardium and myocardium has been identified by single cell transcriptomic analyses. Most of the clusters expressed Wt1, which explains expression in some cardiomyocytes and epicardium later in life [25], suggesting that these few cells with low level Wt1 expression are sufficient to maintain a cardiac progenitor subset from terminal differentiation, which becomes reactivated in cardiac repair.

It is necessary to underline, that the initial proposition of the importance of the epicardium in the formation of fibroblasts was based on experimental studies using avian model systems [36,156] until differentiation of Wt1-expressing EPDCs in cardiac fibroblasts in the mouse has been demonstrated in 2012. By employing a mWt1/IRES/GFP-Cre (Wt1^Cre^) mouse line, Wessels and coworkers proposed that Wt1^+^EPDCs contribute to the majority of cardiac fibroblasts [27]. It has further been shown that epicardial knockout using an inducible *Wt1^C^*^reERT2^ mouse line of serum response factor (Srf) or myocardin related transcription factors (Mrtfs), which function as Srf co-activators, disrupts EPDC migration in development, leading to subepicardial hemorrhage probably due to a reduction in EPDC-derived pericytes which stabilize the coronary vessels [157]. Pericytes are mural cells and are found residing within the basement membrane in microvessels, they are able to differentiate in adipocytes, vascular smooth muscle cells (VSMCs), and myofibroblasts, and consequently modulate the vascular network [158]. However, a clear indication that Wt1 marks pericytes, is still missing until now. This is also because there is no single molecular marker that unequivocally identifies pericytes and distinguishes them from vascular smooth muscle or other mesenchymal cells. In adult mice following myocardial infarction, epicardial specific deletion of Srf or Mrtfs resulted in an improved functional outcome after MI and decreased left ventricular fibrosis [159]. In line with this, Zhang and coworkers suggested that the embryonic epicardium and derived mesenchymal cells were the major source of fibroblasts in endocardial fibroelastosis (EFE), a pathological condition characterized by diffuse profound thickening of the endocardium with abnormal deposition of collagen and elastin predominantly in the left ventricle, often associated with hypoplastic left heart syndrome (HLHS) [160]. The developmental origins and activation of cardiac fibroblasts in response to injury have been reviewed recently [161].

Regarding a possible implication of epicardial-derived Wt1-expressing progenitor cells for cardiac repair, the opinions are diverging. Some studies suggest an important role after myocardial infarction [118,119,162,163,164], while others did not confirm these results [150]. These controversial results derive from the different experimental approaches, staining procedures, and limitations of the Wt1-Cre mouse models used [35,165]. Re-activated epicardium is heterogenous and different from developmental epicardial cells [106], only a few cells in adult epicardium express Wt1 and are reliable targeted by the Wt1Cre lines [35,166].

Epicardial epithelial-mesenchymal transition (EMT) generates the formation of epicardial-derived cells (EPDCs), a population of multipotent mesenchymal cardiac progenitor cells, that may differentiate into various cardiac cell types. The concrete role of Wt1 in this process remained to be elucidated. Martinez-Estrada and colleagues demonstrated that an epicardial specific knockout of Wt1 resulted in a reduction of mesenchymal progenitor cells and their resultant differentiated cell types in the heart. Using immortalized tamoxifen-inducible WT1-knockout epicardial cells, the authors identified direct transcriptional regulation of Snail (Snai1), a master regulator of EMT (reviewed in [167]) and E-Cadherin by Wt1 as the underlying molecular mechanism of EMT. Wt1 activated the Snail promoter while epithelial E-Cadherin appears to be repressed through a double mechanism: first, through direct inhibition by Wt1, second, through repression by Snail, which itself is activated by Wt1 [86]. However, in a study investigating the cardiovascular defects in platelet-derived growth factor receptor (Pdgfr) α-deficient mice, the authors observed a significant increase of Wt1 expression both in Pdgfrα-deficient hearts as well as in small-hairpin RNA PDGFRα-transduced human adult epicardial cells which was not accompanied by an altered expression of E-Cadherin [168]. Accordingly, the effect of WT1 overexpression on E-cadherin expression seems not directly correlated to the effect of WT1 knockdown as described by Martinez-Estrada. Further discrepancies to the findings of Martinez-Estrada emerged from a following study investigating EMT in human epicardial cells. Human adult epicardial cells lost their epithelial character and gained α smooth actin (α SMA) expression when stimulated by transforming growth factor receptor β (TGFβ). This TGFβ-induced EMT was accompanied by a decrease of WT1 and E-Cadherin expression, and an increase of Snail and PDGFRα expression. Similar results were obtained using WT1 knockdown in the human epicardial cells. The contradiction between these data and the findings from Martinez-Estrada and colleagues might be due to the differences in the EMT process of human adult epicardial cells and mouse embryonic epicardial cells [169]. However, Casanova and colleagues established an epicardial-specific knockout model for Snail, which neither demonstrated any cardiac abnormalities nor abnormalities in epicardial EMT, indicating that Snail is simply not required for cardiac epicardial EMT [170]. Using primary murine epicardial cells, Takeichi and colleagues suggested that epicardial EMT is regulated through the bi-directional regulation of Slug (Snai2) by Wt1 and Tbx18 (T-box18). Tbx18 upregulated, Wt1 downregulated Slug by binding to its promoter and affecting its activity [87]. However, no epicardial defects have been reported in Tbx18 knockout or transgenic overexpression models [171], which questions the relevance for Tbx18 in epicardial EMT.

The group of W. Pu did not observe any alterations of E-Cadherin expression in Wt1-deficient epicardium compared to control epicardium. They evidenced in contrast a reduction of Lef1 and Ctnnb1 *(*β-catenin), components of the Wnt/β signaling pathway, as well as decreased Wnt5 and Raldh2 expression in the epicardium of Wt1 knockout animals, suggesting that Wt1 regulates EMT through β-catenin and retinoic acid signaling [172]. In a very nice study, the group of Kispert took advantage of the expression of Wt1 in the mesothelial lining of the heart and analyzed the mechanisms of mesothelial mobilization in cardiac development by inducible genetic lineage tracing. They observed that epicardial mobilization occurs from E12.5 to E14.5 at relatively constant rates. To exclude that proepicardial and eventually myocardial activity of the Cre driver lines influenced extra-epicardial lineages which in turn could affect the epicardium, they activated the Wt1^CreERT2^ driver line in a way that enabled to achieve epicardial recombination at E10.5, when the mesothelial lining has just completed its formation. Using this approach, they demonstrated a functional requirement of platelet-derived growth factor receptor (Pdgfr)α, fibroblast growth factor receptor (Fgfr) 1/2, Hedgehog (Hh), and Smoothened (Smo), but not for Notch, canonical Wnt, and Tgf-β/bone morphogenetic protein (Bmp) signaling in this process [173]. Expression analysis of five epicardial markers, Wt1, Transcription factor (Tcf) 21, Tbx18, Semaphorin (Sema) 3d, and Scleraxis BHLH Transcription Factor (Scx) in development revealed overlapping expression in the proepicardial organ and the epicardium until E13.5, suggesting that epicardium-derived cell fate is specified after EMT [174]. A microarray-based expression analysis of transcriptional changes associated with *Wt1* deletion in *Cre^+^* embryonic epicardial cells and subsequent molecular approaches identified the chemokines Ccl5, Cxcl10, and the interferon regulatory factor Irf7 as transcriptional targets of Wt1 in the heart. Cxcl 10 was shown to inhibit epicardial cell migration and Ccl5 impaired cardiomyocyte proliferation, which could partially explain the reduced number of EPDCs and the thinned myocardium of Wt1-deficient animals. The regulation of Irf7 by Wt1 was proposed to be implied in cardiac repair mechanisms [91]. Transcriptional regulation of bone morphogenetic protein (Bmp) 4 by Wt1 is required for the transition of epicardial cells from a cuboidal morphology to a squamous, flattened cell shape [93]. A very recent study identified the proteoglycan agrin and its receptor dystroglycan, components of the extracellular matrix (ECM) to be required for proper epicardial EMT. Agrin deficiency impaired EMT and disturbed development of the epicardium, also reflected by a down-regulation of Wt1 [175].

Mesothelium, including epicardium, had been shown to contribute to visceral fat [176]. In line with this finding, it had been demonstrated that epicardial progenitors contribute to adipocytes during development, a process termed epicardium to fat transition (EFT). In adult, this programming is quiescent, but can be reactivated by cardiac injury [120]. An elegant study focused on how mechanical stimuli in cardiomyocytes can influence cardiomyocyte cell fates to transdifferentiate into adipose tissue. They demonstrated that Wt1, although not sufficient to provoke the myocyte-to-adipocyte switch, is essential for the conversion process induced by peroxisome proliferator receptor (PPAR)γ. Further, a physical co-interaction of WT1 and PPARγ in the nucleus of myocyte–adipocyte converting cells has been measured, suggesting a cooperative role of the two transcription factors in regulating the adipogenic program [177]. Tang and colleagues reported an up- regulation of Wt1 expression in the pericardial adipose tissue following myocardial infarction. They further characterized Wt1-expressing pericardial adipose-derived stem cells (pADSCs) for cardiac repair. In vitro experiments demonstrated the capability of Wt1+ pADSCs to differentiate into vessel-like structures and contracting cardiomyocytes. In vivo transplantation of Wt1+ pADSCs into infarcted hearts resulted in significant cardiac benefits through Hgf (hepatocyte growth factor) mediated proangiogenic and antiapoptotic effects [178].

Only recently, a function of Wt1 for immune cell regulation in the heart emerged. Wt1 expression in the epicardium seems to be required for the establishment of macrophages originating from fetal yolk sac which reside below and within the epicardium, which later become cardiac resident macrophages [179], implicated in cardiac repair and homeostasis [180,181]. Whether Wt1 acts as a transcriptional regulator of the epicardial program required for fetal yolk sac macrophage recruitment to the heart or if Wt1 impacts recruitment indirectly through its prominent role in the formation of the epicardial structure remains to be further elucidated [179]. *Wt1*^+^ stromal cells in peritoneal, pleural, and pericardial organs, through retinoic acid metabolism, assure homeostasis of resident large cavity macrophages which are involved in tissue repair [182]. In zebrafish, a subpopulation of Wt1b-expressing macrophages could be identified, which contributed to organ repair, especially in the context of cardiac injury [121]. Given our observation that Wt1 is expressed in immune cells in the context of cancer [8], it will be interesting to further identify an implication of Wt1 in immune responses in the context of cardiovascular diseases.

Not much is known how Wt1 is regulated in the heart. Apart from hypoxia and direct transcriptional activation by HIF-1 [100], which are likely to be involved in cardiac development and repair, Hippo signaling components have been proposed to regulate Wt1 expression, epicardial EMT and epicardial cell proliferation and differentiation into coronary endothelial cells [183]. Vieira and colleagues identified an epigenetic mechanism implicating chromatin remodeling of the *Wt1* locus as a critical event in epicardial activity in the developing and adult heart after cardiac injury. They suggested that Wt1 is dynamically controlled by SWItch/sucrose nonfermentable (SWI/SNF) chromatin-remodeling complexes containing Brahma-related gene 1 (BRG1) and Tβ4 [184].

Identification of WT1-modulating factors is of great interest regarding a potential role for cardiac repair in vivo, which is limited due to the lack of techniques to isolate, expand, differentiate, and transfer Wt1+ progenitor cells. However, Wt1 upregulation to enhance cardiac repair might promote tumor growth in patients at risk and should be cautiously monitored. Further research is necessary to delineate the intricacies of modulating WT1 for an optimized therapeutic benefit.

## 5. Conclusions

Wt1 has now been firmly established as a key player in cardiovascular development. It regulates critical steps in heart development, such as formation of the epicardium from the proepicardium, epithelial to mesenchymal transition of epicardial cells, establishment of the coronary vessels, cardiac autonomic nervous system modulation, and the compaction of the ventricles. A very important clinical task remains to establish a profound cardiac evaluation of patients presenting WT1 mutations; *WT1* has long regarded as a gene implicated solely in kidney disorders. We are convinced that WT1 is implicated apart from cardiac diseases also in neurological, ophthalmological, as well as hematological disorders. A function for Wt1 in cardiac repair and regeneration has been proposed based on the finding that it is strongly re-expressed in epicardial, endothelial, and myocardial cells after myocardial infarction. However, to translate these expression patterns in potential therapeutic approaches, more knowledge about the different cardiac cell types and the consequences of the upregulation of WT1 is required. Finally, caution must be taken regarding the tumor-promoting role of this protein in patients susceptible to cancer.

## Figures and Tables

**Table 1:** Wt1-expressing cell types during heart development.

Cell Type	Species	Reference
proepicardial cells	mouse, bird, zebrafish	[23,24,30,31]
epicardial cells	mouse, bird, zebrafish, human	[24,28,30,31,32,33]
endocardial cells	bird, human	[28,34]
subepicardial mesenchymal cells (SEMC), epicardial-derived cells (EPDCs)	mouse, bird	[24,28,31,32]
valvular interstitial cells	bird	[28]
smooth muscle cells	bird	[28]
endothelial cells	bird, mouse, human	[28,31,32,34,35]
fibroblasts	bird, mouse	[27,36]
cardiomyocytes	mouse, human	[26,33,37]

**Table 3:** Wt1-expressing cell types in adult heart.

Cell Type	Species	Reference
epicardial cells	rat, mouse, human	[95,102]
endothelial cells	rat (MI, ischemia), mouse (MI, ischemia)	[95,118]
vascular smooth muscle cells	rat (MI, ischemia)	[95]
cardiomyocytes	mouse (priming with Tβ4, followed by MI, ischemia)	[119]
	mouse	[37]
fibroblasts	mouse (MI, ischemia)	[118]
adipocytes	mouse (MI, ischemia)	[120]
macrophages	zebrafish (cardiac injury)	[121]

## Abbreviations

ACM	Arrhythmogenic cardiomyopathy
αSMA	α smooth actin
AV	Atrio-ventricular
β2AR	β2 adrenergic receptor
BDNF	Brain-derived neurotrophic factor
Bmp	Bone morphogenetic protein
Brg1	Brahma-related gene 1
Ccl5	C-C motif chemokine ligand 5
C-Kit	Tyrosine-protein kinase KIT
CSF-1	Colony-stimulating factor-1
Ctnnb1	β-catenin
Cxcl10	C-X-C motif chemokine ligand 10
E	Embryonic day
EFE	Endocardial fibroelastosis
EFT	Epicardium to fat transition
EMT	Epithelial mesenchymal transition
EPDCs	Epicardial-derived cells
EGFR	Epidermal growth factor receptor
Egr 1	Early growth response protein 1
EPO	Erythropoietin
Ets	ETS proto-oncogene
Fgf	Fibroblast growth factor
Fgfr	Fibroblast growth factor receptor
Gata	Gata-binding protein
HH	Hedgehog
Hif-1	Hypoxia inducible factor 1
HLHS	Hypoplastic left heart syndrome
IGF-1-R	Insulin like growth factor 1 receptor
IGF 2	Insulin like growth factor 2
Irf	Interferon regulatory factor
IR	Insulin receptor
LAD	Ligation of the left coronary artery
LV	Left ventricle
MESCs	Mouse embryonic stem cells
MET	Mesenchymal epithelial transition
MI	Myocardial infarction
Mrtfs	Myocardin related transcription factors
Ncam	Neural cell adhesion molecule
Nfat	Nuclear factor of activated T cells
Nphs1	Nephrin
Nphs2	Podocin
NT	Neurotrophin
P	Postnatal day
pADSCs	Pericardial adipose-derived stem cells
Pax 2	Paired box gene 2
Pdgfa	Platelet-derived growth factor A
Pdgfr	Platelet-derived growth factor receptor
Pecam-1	Platelet and endothelial cell adhesion molecule 1
Ppar	Peroxisome proliferator receptor
PPC	Pericardioperitoneal canal
PPM	Pleuropericardial membrane
Raldh	Retinaldehyde-dehydrogenase
RA	Retinoic acid
RAR	Retinoic acid receptor
RV	Right ventricle
Scx	Scleraxis BHLH transcription factor
Sema	Semaphorin
SEMCs	Subepicardial mesenchymal cells
SHR	Spontaneously hypertensive rat
Smo	Smoothened
Snai1	Snail
Snai2	Slug
Srf	Serum response factor
ST/PE	Septum transversum/proepicardium
SWI/SNF	SWItch/sucrose nonfermentable
Tbx	T-box transcription factor
Tcf	Transcription factor
Tgf-β	Transforming growth factor beta
Tβ4	Thymosin β4
Trf	Telomere repeat binding factor
Trk	Tyrosinkinase receptor
Tubb3	Tubulin beta-3 chain
Vdr	Vitamin D receptor
Vegf	Vascular endothelial growth factor
Vegfr	Vascular endothelial growth factor receptor
Wt1	Wilms’ tumor suppressor 1
Wtip	Wt1 interacting protein
YAC	Yeast artificial chromosome
